# Disease Activity and Progression in Multiple Sclerosis: New Evidences and Future Perspectives

**DOI:** 10.3390/jcm11226643

**Published:** 2022-11-09

**Authors:** Ilaria Del Negro, Sara Pez, Gian Luigi Gigli, Mariarosaria Valente

**Affiliations:** Clinical Neurology Unit, Department of Neuroscience, Udine University Hospital, Piazzale S. Maria della Misericordia, 33100 Udine, Italy

Multiple sclerosis (MS) is a chronic, debilitating, autoimmune-mediated, inflammatory disease of the central nervous system (CNS), in which a combination of inflammation, demyelination and axonal degeneration takes place with extreme highly interpersonal variability.

In recent years, new concepts regarding the mechanisms of inflammation, neurodegeneration, and the onset of disability have emerged. The historical distinction between relapsing and progressive forms of MS has recently been reassessed and compared to a new consensus, in which MS is identified as a continuum and relapsing and progressive phenotypes coexist. Progression and neurodegeneration are present early in the disease course; they occur independently of the MS type and seem to predict the burden of long-term disability, independently from relapses and neuroradiological activity. Since neurodegeneration is strongly associated with worsening disability, the early identification of possible risk factors and signs of progression is essential in making treatment decisions and improving clinical outcomes.

Thus far, many studies have focused on the identification of prognostic risks factors for a better assessment of disease stage and underlying pathophysiology.

The age of onset of MS is a primary factor for the prediction of disease activity. Early-onset MS (EOMS), defined as MS diagnosis between 10 and 18 years, is characterized by high levels of inflammation and a high number of relapses in the first years of disease and infratentorial symptoms, which lead to a high risk of physical disability and cognitive impairment. In this group of patients, EDSS at the onset of the disease seems to be the major prognostic factor for disability. EOMS patients might face a more negative disease course and an early switch to progressive disease compared with adult-onset MS (AOMS) or late-onset multiple sclerosis (LOMS). LOMS manifests a more progressive disease with a minor number of relapses and more severe motor disability; diagnosis is often delayed due to the absence of relapses in the first years. Clinical prognostic factors, leading to a more aggressive disease, are EDSS at diagnosis, new spinal cord lesions, or the contrast enhancement of spinal lesions [1].

The role of spinal lesions as a negative prognostic factor in the course of the disease have largely been investigated and discussed. The recent reviewed MAGNIMS guidelines do not recommend spinal magnetic resonance study for the follow up and monitoring of stable MS patients without spinal cord symptoms. However, Ostini et al. reported a rate of 25% of new asymptomatic spinal cord lesions in stable MS patients, during a median follow up of 17 months. New spinal cord lesions were correlated with the risk of new cerebral or spinal cord lesions and with higher risk of clinical relapses. On the other hand, the presence of new asymptomatic spinal lesions seems to not be correlated with disability progression. Nevertheless, a reduced ability of the spinal cord to compensate neuronal damage should be taken into consideration in the context of the prognostic process [2].

Another aspect to take into account is the role of relapses in disease progression. Disease-modifying therapies reduce relapse rate and seems to slow disability progression, although the quantification of this response is still a matter of debate. Relapse-associated worsening (RAW) is the deterioration of a neurologic function due to an incomplete recovery from a relapse. Relapse and RAW widely impact quality of life and short-term disability, but recent studies did not find an association with long-term disability, progression and clinical outcomes. In the same way, the role of lesion load in brain magnetic resonance imaging (MRI) during the prognosis of the disease is still debated and controversial. Beyond acute inflammation and independently of relapses, new concepts of progression independent of relapse activity (PIRA), progression independent of brain mRi activity and progression independent of relapse and brain MRI activity (PIRMA) have been introduced in light of emerging opinion regarding the presence of underlying mechanisms that are involved in disease and disability progression. PIRA and PIRMA seem to already be present in the early phases of disease and may even occur during first-line treatments; high-efficacy therapy seems to reduce brain atrophy and is therefore thought to have a major impact on reducing disease progression [3]. 

Neuronal loss and brain atrophy have recently been taken into account as markers of neurodegeneration and prognostic factors for developing motor and cognitive disability. Multiple studies indicate that brain volume loss proceeds three to five times more rapidly in patients with MS versus the general population. Although neurodegeneration seems to independently progress from clinical relapses and new MRI lesions, NEDA-3 status (freedom from new relapses, new MRI lesions and clinical disability worsening) is shown to correlate with a lower rate of brain volume loss compared with no-NEDA-3 patients. It is well-recognized that the NEDA-3 target is more likely to be reached with the early administration of high-efficacy therapies, which indirectly lead to a better long-term prognosis in patients treated with high-efficacy therapy as their first treatment option [4].

However, the precise mechanisms underlying neuroinflammation and neurodegeneration involved in disability progression out of clear relapses remain only partially understood. In view of the many new high-efficacy therapeutic strategies, numerous efforts have been made to identify novel biomarkers of disease activity.

Many studies focused on proteins as biomarkers of inflammation and neurodegeneration, which could be involved in diagnostic, prognostic and therapeutic process. Neurofilament light chain (NfL) has arisen as a biomarker of axonal loss and neurodegeneration and, in more recent times, disease activity in MS. A recent study by Kulczyńska-Przybik et al. (2022) analyzed the role of known biomarkers of neurodegeneration and axonal dysfunction in MS patients. A relation between NfL and tau protein was found with demyelination in MRI. NfL has been demonstrated to be a reliable diagnostic biomarker that is able to differentiate between SM patients and controls. The new protein RTN4, which is a potent inhibitor of neurite outgrowth, seems to be less informative in the diagnostic process but accumulates increasing value as demyelination processes become more evident and in the later phases of disease, representing a marker of the altered ability of axonal regeneration. Moreover, RTN4 may induce the secretion of proinflammatory cytokines by macrophages, such as tumor necrosis factor-α and interleukin-1β, thus highlighting the pathogenetic role of these cells in the disease. In addition, RTN4 was found to correlate with immunoglobulin quotient, and thus with inflammatory state and immunological response in MS. All previous biomarkers seem to follow the course of the disease, increasing during worsening and decreasing during stable phases [5]. 

A new emerging frontier of research, in the field of the MS pathogenesis, is the analysis of molecular mechanisms involved in disease onset and progression. One of the promising fields is metabolomics, the study of small molecules expressed in biofluid samples and involved in inflammatory or neurodegeneration activity. Metabolites can be thought as the products of the interaction between genes and environmental stimuli (comprising microbiota), thus allowing a better characterization of the molecular processes and signaling in MS patients. Metabolomics has been proposed as an innovative opportunity to analyze multiple molecules as biomarkers of progression in MS on a large-scale. An increasing interest in the study of metabolic profile of patients with MS and other neuroinflammatory/neurodegenerative diseases is developing. A large metabolomic analysis conducted on serum and cerebrospinal fluid (CSF) demonstrated altered glutathione and nitrogen metabolism in MS patients. These pathways are strongly related to oxidative stress and radical oxygen species (ROS), which seem to be major contributors of neuronal loss, axonal damage and demyelination, and are involved in disease progression and the absence of clearly inflammatory events. ROS are a product of abnormal mitochondrial oxygen metabolism and can play a direct role in damaging neurons and glial cells, as well as activating transcription factors that promote the secretion of inflammatory molecules involved in the pathogenesis of MS (TNF-α, nitric oxide synthase, intracellular adhesion molecule-1). A major expression of these oxidative stress metabolites was found in the progressive phase of MS, but all patients with MS already manifest a progressive increase in oxidative stress since their early stages of disease. Glutamine and glutamate metabolism, leading to brain excitotoxicity, was altered in MS patients. These molecules are necessary for a correct synaptic connection and for the balance between excitatory and inhibitory processes. Glutamate concentration is accurately preserved in serum due to its neuronal excitatory capacity. An altered balance in maintaining glutamate levels in the normal range results in the overexpression of glutamate receptors and excitotoxic injury, causing neuronal loss and glial cells dysfunction. Moreover, glutamate and aspartate levels seem to be higher during relapse phases and even during phases of clinical stability of the disease. Alterations of metabolic pathway, emerging as deeply involved in the neurodegenerative processes recognized in MS, seem to be a new promising tool in the prognostic assessment and in the monitoring of disease progression [6]. 

In MS patients, it is believed that an imbalance between ROS and antioxidant elements exists, leading to structural damages in the central nervous system, and thus neurodegenerative processes. ROS are normally antagonized by the antioxidant capacity of neuronal tissue. In the early stages of disease, it seems that immune activation causes myelin damage through the release of ROS; in fact, the antioxidant capacity of the central nervous system is reduced. A recent study conducted in a cohort of early-stage MS patients demonstrated a significantly higher level of oxidative stress markers in plasma as well as a significantly decreased total antioxidant capacity (TAC), even in the early phases of disease and in clinically stable patients. Therefore, it can be deducted that an increased level of oxidative stress in the contest for a proinflammatory status could be a preclinical sign of progression, suggesting the need of an early intervention to guarantee antioxidant homeostasis [7]. 

In the light of all the previous considerations, any effort should be made to reach a more clear and precise view of the disease stage, inflammatory burden and progression rate for each patient. The prompt identification of disease progression is vital in order to adopt strategies to prevent long-term neurodegeneration and improve prognosis. The individuation of markers or effective tools to detect disease progression is a challenging issue for neurologists, due to the heterogeneity of the disease and the lack of defined criteria for progressive disease. At present, the Expanded Disability Status Scale (EDSS) is the most widely used tool to assess neurological disability; however, numerous limitations of EDSS have been discussed, such as inter-operator variability, the underestimation of upper limb function, vision and cognitive parameters, and low sensitivity at higher values, in which the score mostly corresponds to ambulatory capacity. In order to reduce these limitations, additional instruments are often associated with EDSS in a composite score (nine-hole peg test for manual dexterity or timed 25-feet-walk test for ambulation), but they are not able to quickly identify progression, resulting in a diagnostic delay of the progressive stage of disease. An example of a strategy, developed to facilitate the recognition of disease progression and facilitate the interpretation of complex clinical data, is the MSProDiscuss tool, integrating key symptoms that impact the transition to progressive forms, and algorithms to determine the weight of each parameter in the global progression of disease. This tool collects demographic data, clinical and neuroradiological information for the previous 6 months, evaluates the grade of recovery from any relapse or new activities on MRI and analyzes the impact of symptoms on quality of life and daily activities. Finally, the MSProDiscuss algorithm generates a score that indicates the likelihood of progression for that specific patient (the range varies from 0 to 100 and the risk of progression is estimated as “unlikely”, “possibly” or “likely”). This is a validated tool that can be used to predict the risk of progression, as well as to monitor the trajectory of disease course in the long term [8]. 

In conclusion, MS is a complex and highly variable disease in which both inflammation and neurodegeneration coexist since the early stages of disease. The lack of predictive tests, the scarcity of biomarkers of disease activity, and consequently, the difficulty to recognize precise phases of disease required for a consensus on phenotypic classification are only some of the aspects that still need to be investigated in future research. New high-efficacy therapies, although effective in contrasting neuroinflammation and neurodegeneration, especially in the initial phase of disease, are insufficient to arrest their underlying processes. Mechanisms of neurodegenerations, as well as alterations of energetic and mitochondrial functioning and accelerated biological aging, are still largely unknown, and these all take part in the pathogenesis of MS. Neurodegeneration, represented by axonal loss, brain atrophy and specific fluid biomarkers, is strongly correlated with functional and cognitive long-term disability. Neurologists’ efforts should therefore be directed toward a more accurate and personalized MS care and rapid identification of hidden disease progression. Different biomarkers and more advanced technologies, such as metabolomics, may open new frontiers in understanding this silent progression and offer novel potential strategies to counteract it. If pathological mechanisms still need to be clarified, promising data regarding future prognostic factors are emerging from recent studies for a better insight into the disease course of MS.
